# Graphene Oxide Nanoscale Platform Enhances the Anti‐Cancer Properties of Bortezomib in Glioblastoma Models

**DOI:** 10.1002/adhm.202201968

**Published:** 2022-11-11

**Authors:** Paul S. Sharp, Maria Stylianou, Luis M. Arellano, Juliana C. Neves, Alfredo M. Gravagnuolo, Abbie Dodd, Katharine Barr, Neus Lozano, Thomas Kisby, Kostas Kostarelos

**Affiliations:** ^1^ Nanomedicine Lab Faculty of Biology, Medicine & Health National Graphene Institute University of Manchester AV Hill Building Manchester M13 9PT UK; ^2^ Catalan Institute of Nanoscience and Nanotechnology (ICN2) Campus UAB, Bellaterra Barcelona 08193 Spain; ^3^ Present address: Medicines Discovery Catapult Alderley Park, Mereside Macclesfield SK10 4TG UK

**Keywords:** 2D materials, chemotherapy, glioblastoma, graphene, nanomedicine

## Abstract

Graphene‐based 2D nanomaterials possess unique physicochemical characteristics which can be utilized in various biomedical applications, including the transport and presentation of chemotherapeutic agents. In glioblastoma multiforme (GBM), intratumorally administered thin graphene oxide (GO) nanosheets demonstrate a widespread distribution throughout the tumor volume without impact on tumor growth, nor spread into normal brain tissue. Such intratumoral localization and distribution can offer multiple opportunities for treatment and modulation of the GBM microenvironment. Here, the kinetics of GO nanosheet distribution in orthotopic GBM mouse models is described and a novel nano‐chemotherapeutic approach utilizing thin GO sheets as platforms to non‐covalently complex a proteasome inhibitor, bortezomib (BTZ), is rationally designed. Through the characterization of the GO:BTZ complexes, a high loading capacity of the small molecule on the GO surface with sustained BTZ biological activity in vitro is demonstrated. In vivo, a single low‐volume intratumoral administration of GO:BTZ complex shows an enhanced cytotoxic effect compared to free drug in two orthotopic GBM mouse models. This study provides evidence of the potential that thin and small GO sheets hold as flat nanoscale platforms for GBM treatment by increasing the bioavailable drug concentration locally, leading to an enhanced therapeutic effect.

## Graphical abstract

Small, thin graphene oxide (GO) nanosheets undergo time‐dependent translocation and retention within the brain tumor microenvironment. GO nanosheets also offer a biocompatible, high‐surface‐area platform for the efficient non‐covalent complexation of chemotherapeutic agents. Loading GO with the proteasome inhibitor bortezomib (BTZ) provides improved efficacy and anti‐cancer activity following intratumoral administration in two orthotopic glioblastoma models.

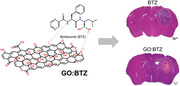

## 1 Introduction

Glioblastoma multiforme (GBM; WHO grade IV glioma) is the most aggressive and prevalent form of adult primary cancer of the central nervous system.^[^ ^1^ ^]^ The standard of care is surgical resection (if tumor location allows), followed by radiotherapy with complementary cycles of systemically administered temozolomide (TMZ) chemotherapy. Despite this combinatory approach, the prognosis of GBM is invariably a terminal disease with a median survival of 15–18 months, and 5‐year survival of less than 10%.^[^ ^2^ ^]^ The employment of novel therapeutic approaches including convection‐enhanced delivery, targeted therapies, monoclonal antibodies, and tumor‐treating fields has not yet had a major impact on this poor prognosis.^[^ ^3^, ^4^, ^5^, ^6^ ^]^

GBM has proved challenging to treat due to the highly aggressive and invasive nature of tumor cells and their unique location in the brain, protected by the blood‐brain barrier (BBB).^[^ ^7^, ^8^ ^]^ Despite numerous efforts to increase the permeability of the BBB to chemotherapy,^[^ ^9^ ^]^ key limitations such as the pharmacokinetic profile of chemotherapeutic agents, along with systemic toxicity have compromised the efficacy of GBM treatments. These challenges have led to an urgent need to develop new strategies that can achieve higher and more localized therapeutic concentrations at the tumor site. One of those strategies is the direct administration of chemotherapy into the tumor parenchyma or resection cavity. However, the rapid clearance kinetics and diffusion away from the tumor or the rapid enzymatic processing and degradation of the therapeutic agents, have led to compromised therapeutic efficacies. Moreover, such effects pose neurological and systemic safety concerns and have thus far required the application of complicated administration methods through an implanted catheter or dissolvable implants, still with varied efficacy reported.^[^ ^10^, ^11^ ^]^ Alternatively, the use of nanomaterials to deliver drugs directly into the tumor can increase the therapeutic agent concentration achieved locally, but also prolong localized high concentrations and provide much‐needed drug stability.^[^ ^12^, ^13^, ^14^, ^15^ ^]^

The unrivaled available surface area of small, thin graphene oxide (GO) nanosheets provides a platform for increased loading capacity of bioactive molecules and can allow excellent colloidal dispersibility in physiological fluids, with a proven bio‐ and neuro‐compatibility profile.^[^ ^16^, ^17^, ^18^, ^19^, ^20^ ^]^ Several efforts have been made to utilize GO as a delivery platform for anti‐cancer agents by either covalent attachment or non‐covalent interactions and in combination with photothermal effects.^[^ ^20^, ^21^, ^22^, ^23^, ^24^, ^25^, ^26^ ^]^ We previously demonstrated that GO nanosheets are able to localize throughout the tumor volume using a human glioblastoma model, both in vitro (3D spheroids) and in vivo (orthotopic xenograft model).^[^ ^27^ ^]^ Taking advantage of the translocation of GO exclusively within the GBM volume and recognizing the limited activity and diffusivity of locally administered small molecules, we have hypothesized that GO sheets could be a promising flat platform to achieve transport, localization, and retention of chemotherapeutic agents intratumorally.

The persistently poor prognosis of GBM patients undergoing standard‐of‐care treatment with TMZ chemotherapy highlights the necessity to explore non‐standard chemotherapeutics. High content screening of patient‐derived cell lines revealed that bortezomib (BTZ; Velcade) was a non‐standard chemotherapeutic agent that showed potent cytotoxic activity across a panel of GBM cell lines, multiple times more effective than TMZ.^[^ ^28^ ^]^ BTZ is the first proteasome inhibitor approved by the US Food and Drug Administration (FDA) for multiple myeloma and mantle cell lymphoma,^[^ ^29^ ^]^ while further demonstrating promising antitumor activity in various cancers including colorectal, pancreatic, and lung cancer.^[^ ^30^ ^]^ However, in the clinic, intravenous administrations of BTZ have shown limited therapeutic efficacy in GBM with the boric acid groups compromising the blood stability of the drug, exhibiting poor BBB translocation, and leading to frequent adverse effects.^[^ ^31^, ^32^, ^33^, ^34^, ^35^, ^36^ ^]^ Preclinical investigations have shown modest improvement of therapeutic efficacy with direct administration to GBM, however, this required continual infusion with a surgically implanted osmotic mini‐pump further limiting the ease of clinical applicability.^[^ ^37^ ^]^ Based on such reported knowledge, we selected BTZ as a suitable potent candidate chemotherapeutic agent for intratumoral (GBM) transport via the GO‐based flat nanomaterial platform.

In this work, we first investigated the distribution kinetics of intratumorally injected GO in vivo using orthotopic mouse GBM models, and then non‐covalently complexed and characterized a GO:BTZ nanoscale complex system. The successful complexation of GO with BTZ demonstrated high drug‐loading capacity, and more importantly, preservation of the drug's biological activity. We evaluated systematically its cytotoxic activity in vitro and in vivo, utilizing two GBM mouse models (orthotopic xenograft and orthotopic syngeneic model). A single low‐volume intratumoral administration of GO:BTZ resulted in significant tumor tissue necrosis and enhanced activity compared to free drug.

## 2 Results

### 2.1 GO Localizes Throughout the GBM Tumor Volume In Vivo

**Figure 1:**
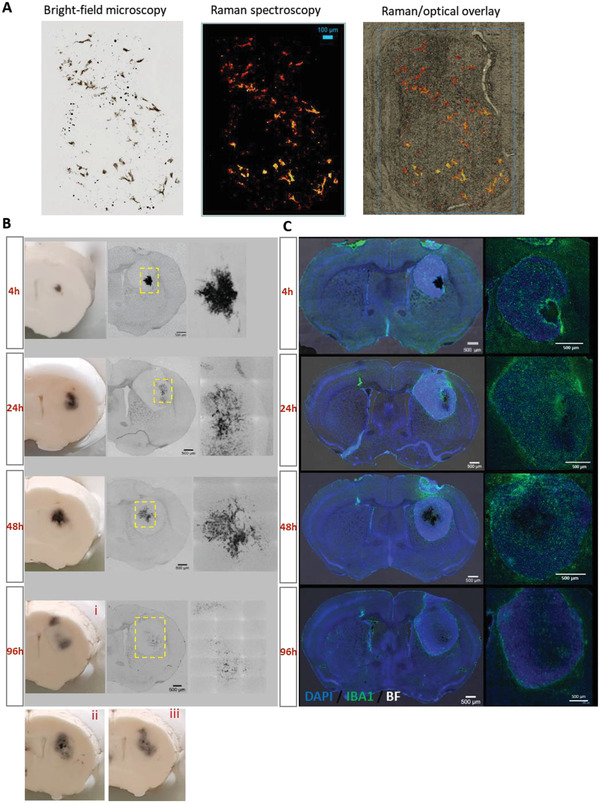
Time‐course of graphene oxide nanosheet distribution in orthotopic U87 gliomas. Athymic nude mice were implanted with 1 × 10^5^ U87‐luc cells into the right striatum. To assess penetrance, 1 µg of GO was delivered intratumorally at 12 days post inoculation (*n* = 12) and brains were harvested at 4, 24, 48, and 96 h post GO delivery (*n* = 3 per group). A) Co‐localization of the GO Raman signal and black matter observed under bright‐field imaging in brain sections. B) Representative images of the whole brain and corresponding 20 µm sections showing the redistribution of intratumoral GO over time (scale bar 500 µm). For the 96 h time‐point, i–iii) images show the presence of GO in an anterior‐posterior (rostral‐caudal) direction. C) Representative epifluorescence images of the whole brain and tumor region showing the distribution of intratumoral GO in relation to DAPI staining and IBA1+ macrophage/microglia cells. Scale bar = 500 µm.

Following our previous observations of extensive GO nanosheet distribution throughout U87‐MG tumors in vivo,^[^ ^27^ ^]^ we initially utilized the U87 model for a more dynamic evaluation of GO translocation within the GBM tissue over time. Human U87‐luc cells were implanted intracranially in athymic mice and injected at the same site with 1 µl GO (1 mg ml^−1^) alone, 12 days following cell inoculation. GO distribution was evaluated histologically at 4, 24, 48, and 96 h post intratumoral injection (**Figure** 1). First, the optical signature of GO (identified as dark material) within the histological specimen was validated with Raman spectroscopy showing co‐localization of GO Raman signature with the observed black matter (Figure 1A). Between 4 and 96 h post‐intratumoral injection, we observed a significant spread of GO from the well‐localized point of the injection site to a more diffuse pattern throughout the U87 tumor area over time (Figure 1B,C). In addition to the observed lateral spread, GO was also identified to translocate both rostrally and caudally by 96 h post‐administration (Figure 1B (i)–(iii)). Importantly, we did not identify any GO outside the tumor border within the healthy brain tissue. In agreement with our previous report that described the interaction and internalization of GO by macrophages and microglia (IBA1+ cells) within the U87 GBM microenvironment,^[^ ^27^, ^38^ ^]^ we further observed a strong IBA1+ signal at the initial injection site at 4 h which appeared to undergo redistribution alongside the spread of the nanomaterial (Figure 1C).

### 2.2 Preparation and Characterization of Non‐Covalent GO:Bortezomib (BTZ) Complexes

**Figure 2:**
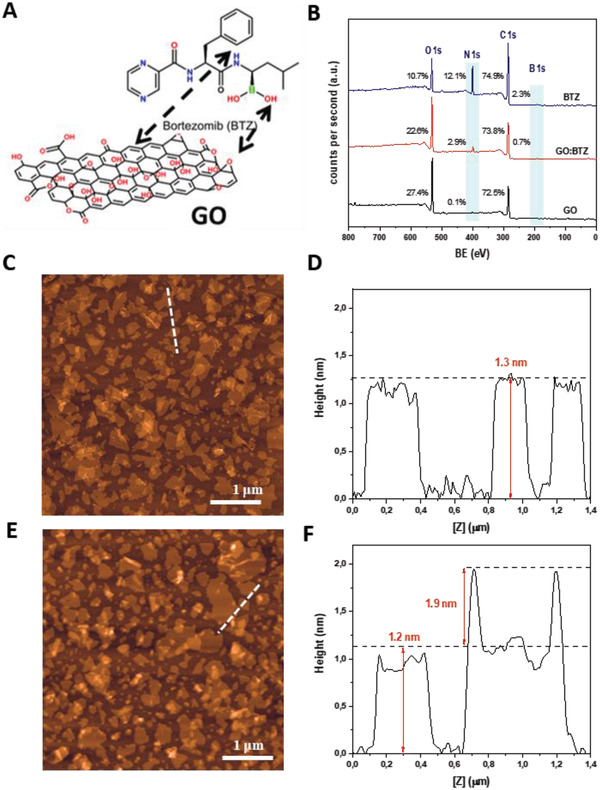
Physicochemical characterization of GO_c_ and GO:BTZ complex. A) Schematic illustrating GO:BTZ complex and predicted chemical interactions. B) XPS survey analysis. C,D) AFM height images and cross‐section analysis of GO and E,F) GO:BTZ complex. Measurements were performed in triplicate.

Given the spatial distribution of GO nanosheets throughout the tumor volume and their retention within the tumor, we reasoned that complexation of the nanomaterial with a chemotherapeutic drug such as BTZ may improve the localization, persistence, and activity of the drug following direct (intratumoral) administration. BTZ molecules were mixed with GO at a 10:6 GO:BTZ (weight ratio) in an aqueous solution (1 mg mL^−1^, GO). BTZ was expected to be complex onto the GO lattice primarily through *π*–*π* stacking interactions between the aromatic rings, with further potential for hydrogen bonding among the oxygen‐containing groups on the GO and the polar groups of BTZ (**Figure** **2**A).^[^ ^39^ ^]^ GO alone control (GO_c_) was also prepared using the same protocol as per the GO:BTZ complex for the purpose of characterization.

To quantify the BTZ loaded onto GO sheets, the concentration of BTZ on the complex was calculated indirectly by serial washing and centrifugation steps in water (as shown in Figure S1A, Supporting Information). The final concentration of BTZ loaded on the GO:BTZ complex was found to be 0.17–0.19 mg BTZ per 1 mg GO (Figure S1B, Supporting Information) as determined by UV–vis using a BTZ concentration standard curve in water (Figure S1C,D, Supporting Information). Complex formation was validated by the XPS survey spectrum for the complex which exhibited signals corresponding to carbon, oxygen, nitrogen, and boron (Figure 2B). The strong nitrogen peak at 400 eV and the weak boron contribution at 191 eV provided further evidence of the absorption of BTZ onto the GO surface. Next, we evaluated BTZ release from the GO:BTZ complex (in water) at 4 and 24 h post‐complex formation using UV–vis based quantification. The total concentration of BTZ released over 24 h was found as 1 µg ml^−1^ (per 1 mg ml^−1^, GO) which indicated high stability of the GO:BTZ complex (Figure S2, Supporting Information).

GO_c_ displayed the characteristic topology of isolated monolayers with an average height of 1.2–1.3 nm by AFM (Figure 2C,D),^[^ ^40^ ^]^ whereas for GO:BTZ complexes, the presence of several bright areas with heights of 1.9 nm suggested the immobilization of BTZ molecules onto the GO basal plane with uniform distribution and without evidence of structural damages (Figure 2E,F and Figure S3A,B, Supporting Information). In the Raman spectra of GO_c_ and GO:BTZ (Figure S3C, Supporting Information) the characteristic D (1327 cm^−1^) and G band (1599 cm^−1^) of graphitic materials were identified.^[^ ^41^ ^]^ When BTZ was loaded on GO, additional Raman peaks attributed to boronic acid compounds were clearly detected,^[^ ^42^ ^]^ indicating the decoration on the GO surface without disturbing the graphitic lattice. In addition, the G‐band was down‐shifted by 4 cm^−1^ with respect to GO_c_ suggesting the n‐doping of the material by the BTZ moiety.^[^ ^43^ ^]^

XRD analysis by analyzing the crystalline phase and interlayer distance for both GO_c_ and GO:BTZ complex, showed a range of 2*θ* from 5° to 40° (Figure S3D, Supporting Information). The XRD patterns confirmed the chemical oxidation of graphite and complete formation of GO by the appearance of a new diffraction peak at 2*θ* = 11.2° and no residual reflection of the graphite plane at 2*θ* = 26.6°. The increase in the interlayer distance was attributed to the uptake of oxygen‐containing functional groups and water molecules between GO layers.^[^ ^44^ ^]^ Furthermore, for the GO:BTZ complex the shift of the characteristic peak of GO from 11.2° to 9.9° with a slight increase in the basal spacing evidencing the intercalation of BTZ molecules between GO sheets. The spectrum of GO:BTZ complex by FTIR spectroscopy was almost the same as the GO_c_ revealing the existence of OH, C=O, C=C, and C–O–C functional groups, with new contributions at 1527 and 1193 cm^−1^ ascribed to C=C/C=N and C–B stretching respectively,^[^ ^45^ ^]^ as well as an increase of the peaks at around 2850–2950 cm^−1^ related to C–H vibrations (Figure S3E, Supporting Information). Interestingly, the shift of the C=O peak in the complex in comparison with parent BTZ suggests the hydrogen bonding between both entities as reported previously in the formation of GO and doxorubicin complexes.^[^ ^46^ ^]^

Further characterization was obtained by dynamic light scattering (DLS) and zeta potential measurements.^[^ ^47^ ^]^ DLS was exclusively used to evaluate the bulk colloidal characteristics of the suspension over time (Figure S3F, Supporting Information). The surface charge of GO nanosheets before and after BTZ complexation remained negative. Generally speaking, there were no significant changes after 7 days for both size and surface charge of the complexes in water suggesting the formation of a colloidally stable complex. Furthermore, we evaluated the bulk colloidal characteristics of the GO_c_ and GO:BTZ complexes suspended in serum (10% FBS) for 24 h (Figure S4, Supporting Information). The bulk colloidal properties of both GO_c_ and GO:BTZ remained similar and in suspension, with some larger agglomerates forming by 24 h in serum as observed by AFM, presumably due to the serum protein coating of the complex. Taken together, these data confirm the non‐covalent complexation of BTZ on the surface of GO nanosheets and the formation of a stable complex.

### 2.3 GO:BTZ Complex Retains Cytotoxic Activity In Vitro

**Figure 3:**
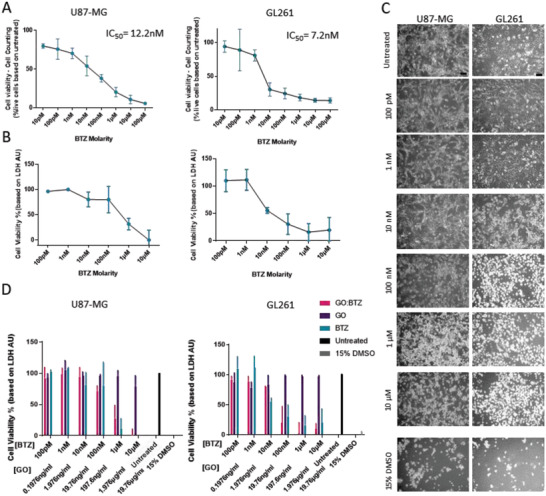
GO:BTZ retains cytotoxic potential in vitro for both GL261 and U87‐MG cells. A) Cell viability graphs of U87‐MG (left side) and GL261 (right side) 24 h after treatment with free BTZ at 10 pM to 100 µM determined by live cell counting and corroborated by B) LDH assay. Data presented as mean ± SD % cell viability relative to control (*n* = 3). C) Representative bright field images taken 24 h post‐treatment. Scale bar, 100 µm. D) Cell viability of U87‐MG (left side) and GL261 (right side) 24 h post‐treatment with GO:BTZ complex, free BTZ, GO_c_, or 15% DMSO. Data presented as mean ± SD % cell viability relative to control (*n* = 3). No statistical significance between the BTZ and GO:BTZ groups (Two‐way ANOVA, Tukey's multiple comparison test).

To evaluate the cytotoxicity of BTZ complexed with GO in vitro, we used a patient‐derived GBM cell line (U87) and a mouse‐derived GBM cell line (GL261).^[^ ^28^, ^48^, ^49^ ^]^ Cell counts using Trypan blue exclusion were performed demonstrating the sensitivity of both GBM cell lines to BTZ with IC_50_ = values of 7.2 nM and 12.2 nM for GL261 and U87‐MG respectively (**Figure** **3**A). This observation was also supported by the LDH assay (Figure 3B) and cell morphology analysis (Figure 3C). To ensure that GO complexation with BTZ did not inhibit BTZ function, we treated U87‐MG and GL261 glioma cells with increasing doses of GO:BTZ (based on BTZ molarity) under the same conditions as previously described. GO:BTZ and BTZ alone showed comparable viability curves without significant differences (Figure 3D). In contrast, GO_c_ showed no impact on cell viability, demonstrating that the cytotoxic activity is attributed to the presence of BTZ on the surface of the GO:BTZ complex. Overall, these results demonstrate that the complexation of BTZ through non‐covalent interaction with GO does not inhibit its biological (cytotoxic) activity in vitro.

### 2.4 GO:BTZ Complex Retains Cytotoxic Activity in U87 Gliomas In Vivo

**Figure 4:**
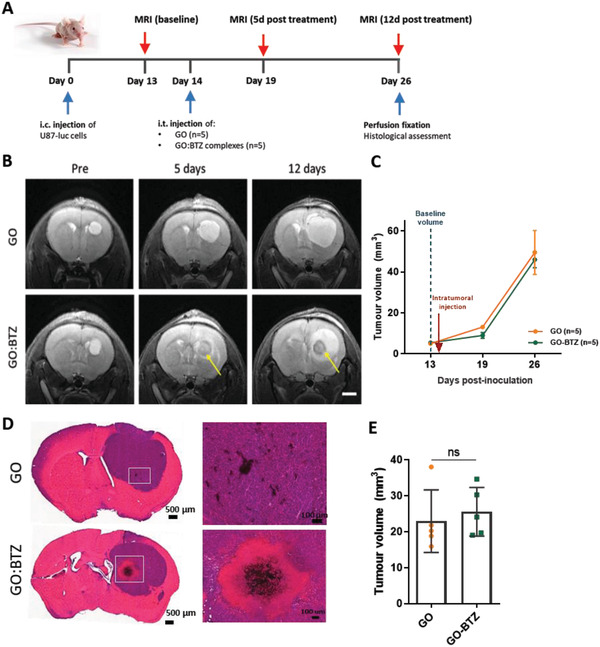
Evaluation of the biological activity of GO:BTZ in vivo in U87 gliomas. (A) Experimental schematic; Athymic nude mice were implanted with 1 × 10^5^ (1 µl) U87‐luc cells into the right striatum. MRI was conducted at 13 days post inoculation for a pre‐treatment (baseline) and 5 and 12 days following intratumoral delivery of GO_c_ (1 µl, 0.9 mg mL^−1^; *n* = 5) or GO:BTZ (0.175 mg ml^−1^, BTZ, *n* = 5). B) Coronal T_2_‐weighted MRI images showing the progression of U87 glioma growth and the effect of intratumoral delivery of GO_c_ (upper panels) and GO:BTZ (lower panels), 5 and 12 days post‐treatment. Arrows highlight areas of hypointensity within the tumor following GO:BTZ treatment that is not observed following GO delivery. Scale bar, 2 mm. C) Volumetric quantification of U87 tumors from MRI scans of GO_c_ (*n* = 5) and GO:BTZ (*n* = 5) treated mice, after subtracting the hypointensity area. Data are presented as mean tumor volume ± S.E.M. No statistical significance between the GO_c_ and GO:BTZ groups (Two‐way analysis of variance‐ANOVA, Sidak's multiple comparisons test). D) Representative H&E images showing the effect of intratumoral delivery of GO_c_ and GO:BTZ on day 12 post‐treatment. Scale bars: 500 µm and 100 µm. E) Quantification of tumor volume based on H&E data after correction for the necrotic zone. Data presented as mean ± SD. No statistical significance after the unpaired Student's *t*‐test.

To further evaluate the cytotoxic activity of the GO:BTZ complexes we initially utilized an orthotopic xenograft model of glioblastoma.^[^ ^50^ ^]^ U87‐luc cells were injected into the striatum of athymic mice and tumors were allowed to establish for 14 days before mice received intratumoral injection of GO_c_ or GO:BTZ (at 0.175 µg BTZ) (**Figure** **4**A). Anatomical *T*_2‐_weighted MRI was conducted at 13 days post inoculation (pre‐treatment) to establish the size and position of the tumor, then again at days 5 and 12 following intratumoral injection. No change, in contrast, was observed in mice treated with GO_c_, whereas a clear impact on the tumor was observed as a defined hypointensity extending from the site of injection in the GO:BTZ treated group, which is suggestive of drug‐induced necrosis (Figure 4B). While no significant change in the tumor volume was calculated (Figure 4C), further histological analysis of this phenomenon present 12 days after treatment, provided consistent evidence across all mice of a GO:BTZ‐induced necrotic zone which was largely devoid of nuclei (Figure 4D and Figure S5, Supporting Information). Despite this prominent effect, endpoint tumor growth was not significantly inhibited by GO:BTZ delivery (Figure 4E), potentially due to the use of a low‐volume approach in a large established tumor.

To understand the kinetics of this GO:BTZ‐induced cytotoxic response we further evaluated this effect at earlier time points (4, 24, and 96 h) following administration of either the GO:BTZ complex or the equivalent dose of BTZ (Figure S6A, Supporting Information). The presence of this cytotoxic core extending from the injection site became apparent by 24 h post‐injection and further developed over the next 48 h consistent with the kinetics for the translocation of the material observed previously (Figure S6B, Supporting Information). Notably, this effect was not visible in the free BTZ‐treated tumors. Taken together, these data provide proof of concept for GO as a delivery system for chemotherapy drugs in an in vivo GBM model and warrant further investigation.

### 2.5 GO:BTZ Complex Exhibits Higher Cytotoxic Activity in an Aggressive Immunocompetent Tumor Model

As the xenograft model did not illustrate a complete and sustained tumor clearance after GO:BTZ intervention, we decided to explore this response further in an immunocompetent syngeneic model. The murine GL261 glioblastoma model exhibits a more rapid tumor growth, retains an intact adaptive immune system and thus more closely recapitulates the cellular populations and invasive nature of clinical GBM^[^ ^51^ ^]^ (Figure S7, Supporting Information). Furthermore, based on our in vitro cytotoxicity assays, GL261 cells show increased sensitivity to BTZ compared to U87 (Figure 3A–C). We initially sought to confirm that the effects previously observed in U87 xenograft could be reproduced in this syngeneic model. GL261 cells were inoculated in the striatum and on day 9 after implantation, mice were injected intratumorally with GO_c_, free BTZ, or GO:BTZ (at 0.175 µg BTZ) (Figure S8A, Supporting Information). Histological analysis of the brains on day 6 after treatment illustrated that GO:BTZ treatment induced a pronounced tumor clearance effect extending outward from the initial injection site (Figure S8B, Supporting Information). Staining of nuclei with DAPI confirmed that this cytotoxic effect was associated with necrosis with the absence of nuclei within the treated tumor area (Figure S8C, Supporting Information). This effect was not detected in the free BTZ or GO_c_‐treated tumors and this is further corroborated by the measurement of tumor volumes which demonstrated that GO:BTZ treatment significantly reduced the tumor volume compared to these two control groups (Figure S8D, Supporting Information).

**Figure 5:**
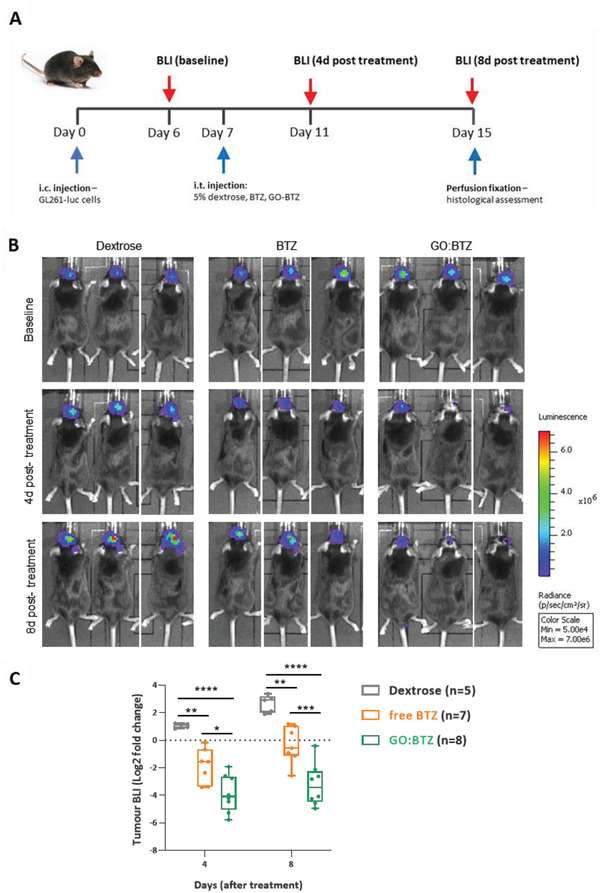
Enhanced tumor‐suppressing activity of GO:BTZ in GL261 glioma. A) Experimental schematic for the in vivo analysis. C57Bl/6 mice were implanted with 5 × 10^4^ (1 µl) GL261‐luc cells into the right striatum. BLI was conducted at 6 days post inoculation (i.c) for a pre‐treatment (baseline) and 4 and 8 days following intratumoral (i.t.) delivery of 5% dextrose (*n* = 5), free BTZ (*n* = 7) or GO:BTZ complex (7 days post i.c.). B) Representative bioluminescence images of GL261 mice at baseline (6 days post i.c.), and 8 days following intratumoral delivery of 5% dextrose, free BTZ, or GO:BTZ. C) Fold change in bioluminescence relative to the pre‐treatment baseline across all treatment groups (dextrose, free BTZ, or GO:BTZ) on 4 and 8 days post‐treatment. Two‐way ANOVA, Sidak's multiple comparisons test (* *p* ≤0.05, **p ≤ 0.01, ***p ≤ 0.001, ****p ≤ 0.0001).

**Figure 6:**
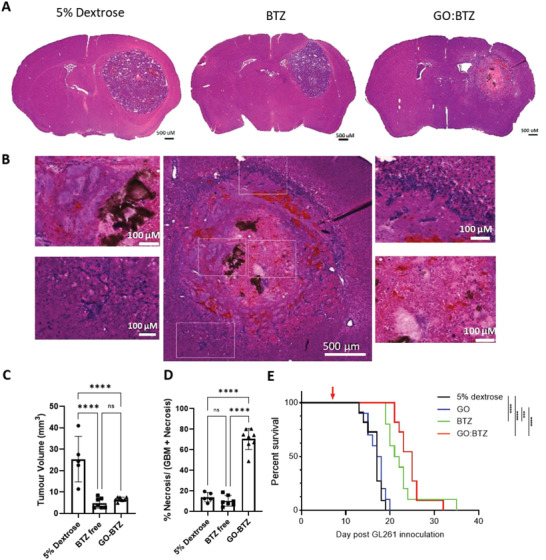
Elevated cytotoxic activity of GO:BTZ in GL261 glioma mice. A) Representative H&E images across treatment groups 8 days post all treatment (5% Dextrose [*n* = 5], free BTZ [*n* = 7], and GO‐BTZ complex [*n* = 7]). Scale bar, 500 µm. B) High magnification H&E images from GO‐BTZ treated brain tumor (8‐day post‐treatment). Scale bar, 500 µM. Zoomed images illustrating different areas of GO‐BTZ treated tumor. Scale bar, 100 µM. C) Tumor volume at 8 days post‐treatment with 5% dextrose (*n* = 5), GO‐BTZ (*n* = 7), and free BTZ (*n* = 7). D) Necrotic area as a percentage of GL261 tumor area determined via orbit image analysis. Data presented as mean ± SD. Ordinary one‐way ANOVA, Tukey's multiple comparisons test (****p ≤ 0.0001). E) Survival analysis of GL261‐luc bearing mice treated with 5% dextrose, GO, free BTZ, or GO:BTZ complex (*n* = 10–11). Day of treatment highlighted with red arrow. Statistical differences shown in index. Log rank (Mantel–Cox) test (***p ≤ 0.001, ****p ≤ 0.0001).

We followed this up with a longitudinal measurement of the response using bioluminescence imaging. Mice were again inoculated with GL261‐luc cells followed by intratumoral intervention on day 7 post‐inoculation (**Figure** **5**A). There was a clear and significant reduction in bioluminescence signal in mice treated with GO:BTZ compared to dextrose vehicle and free BTZ groups on both day‐4 and day‐8 post intratumoral injections (Figure 5B,C). In agreement with in vivo imaging, a remarkable area of tumor necrosis in GO:BTZ treated tumors was further observed by histological analysis of brains from day 8 post‐injection (**Figure** **6**A,B). While tumor volume reconstruction (without correction for the necrotic area) did not show any significant difference between free BTZ and GO:BTZ groups (Figure 6C), using tissue structure‐based classification (Figure S9, Supporting Information)^[^ ^64^ ^]^ we confirmed that the percentage of necrosis was significantly higher in the GO:BTZ group compared to free BTZ (Figure 6D) demonstrating an enhanced cytotoxic effect. Finally, we conducted a further study to understand the longevity of this treatment response. As before, treatment with GO:BTZ significantly reduced the bioluminescence signal by day 4 post‐treatment (Figure S10A,B, Supporting Information) which translated to an increase in median survival from 17 days in the vehicle control group, to 25 days in the GO:BTZ treated group which was also increased beyond that achieved by free BTZ (21.5 days) (Figure 6E). Importantly, GO:BTZ‐treated animals showed no signs of toxicity in relation to bodyweight changes during the acute treatment phase (Figure S10C, Supporting Information) and maintained their weight longer than animals in the control treatment groups (Figure S10D, Supporting Information). Histological analysis of mice at the time of sacrifice illustrated that tumor growth at the treatment site (striatum) remained relatively suppressed, however outgrowth of tumor tissue superficial to this in the uninjected area within the cortex (originating from the needle tract) was able to progress (Figure S10E, Supporting Information). Notably, an amount of material did appear to persist in the necrotic area induced by GO:BTZ, which may limit its translocation in comparison to bare GO and restrict the capacity for GO:BTZ to reach tumor areas further from the initial injection site (Figure S10E, Supporting Information and Figure 6B). While these results are encouraging, the observation that the animals still eventually succumb to the tumor burden even after GO:BTZ treatment highlights limitations associated with single intratumoral injection and warrants further optimization of this nanomaterial‐enabled treatment strategy.

## 3 Discussion

In this study, we utilized GO nanosheets non‐covalently complexed with BTZ as a novel, single dose, intratumorally administered therapy, which achieved an enhanced anti‐cancer activity compared to the free drug in two orthotopic mouse models of glioblastoma.

Following the administration of GO nanoparticles into a human U87 xenograft orthotopic mouse model we observed an outstanding time‐dependent diffusion effect throughout the tumor area without exiting the tumor border, confirming our previous findings.^[^ ^27^ ^]^ The high penetrance of this small, thin (1–2 nm thick) nanomaterial throughout the tumor area highlighted its potential as a drug‐delivery system. Several studies have previously investigated graphene‐based materials for the loading and delivery of chemotherapeutics including doxorubicin, cisplatin, and BTZ.^[^ ^39^, ^52^, ^53^, ^54^, ^55^, ^56^ ^]^ The majority of these studies investigated the effects of their graphene‐based delivery system in 2D or 3D cell cultures that do not recapitulate the complex tissue and cellular interactions of these nanomaterials in the in vivo setting. One study did explore intravenous administration of transferrin functionalized GO nanoparticles for doxorubicin delivery in a C6 glioma rat model.^[^ ^57^ ^]^ However, the administration of multiple doses was necessary to achieve a modest tumor inhibition due to minimal accumulation of drug at the tumor site with the majority of injected dose accumulating in off‐target organs. This highlights the challenges associated with systemic administration for intracranially located tumors. Indeed, while intracranial administration is not the primary drug administration approach currently used in the clinic it has been identified as a potential alternative approach with clear benefits in regards to avoiding the off‐target side effects,^[^ ^37^, ^58^, ^59^ ^]^ and could be further improved using nanoparticles that distribute widely throughout but are retained within the tumor as we observed here. Thus, we rationally utilized GO as a delivery platform to introduce a non‐classical chemotherapeutic following simple intratumoral administration.

The dose of bortezomib in the present study is orders of magnitude below that used for systemic administration in other preclinical investigations (30–40 µg)^[^ ^37^, ^60^ ^]^ and was able to achieve efficacy at a lower dose than was previously reported for intratumoral administration of BTZ in orthotopic GBM models highlighting the advantages of utilizing a nanoscale delivery system.^[^ ^37^, ^61^ ^]^ BTZ has previously been encapsulated in other nanoscale systems, such as CuS/carbon dot nanocomposites or dendrimers aiming for targeted delivery following systemic administration into different types of cancers, including GBM, again requiring relatively high doses due to limited tumor localization.^[^ ^61^, ^62^, ^63^ ^]^ In addition to the doses required, one advantage of a GO nanosheet‐based approach is the rapid non‐covalent complexation of BTZ which maintained biological activity while allowing for gradual detachment of the drug from GO locally throughout the tumor area where GO has translocated. Furthermore, in this study, we identified differences in sensitivity between U87 and GL261 orthotopic models, which highlights the requirement to consider different chemotherapeutics for a heterogeneous patient population, as is the case in human GBM. Indeed, the versatility of exploiting non‐covalent interactions of drugs with GO nanosheets provides significant advantages when considering adaptation in this scenario. We hypothesize that GO could be used as a universal platform to interact and deliver various drugs or combinations to improve therapeutic responses or rapidly adapt to treatment‐resistant tumors.

Despite the enhanced anti‐cancer effects achieved here, the eventual overgrowth of residual tumors beyond the injected treatment site with this approach is a limitation that is shared by alternative preclinical and clinical locally applied therapeutic approaches in GBM. Future investigations may be able to further resolve this through MRI‐guided intratumoral injections to ensure more effective tumor targeting at different sites. In addition, the current nano‐chemotherapeutic approach described here could be further evaluated in combination with other therapies, or with adaptation for the post‐resection environment that could ultimately provide a more long‐lasting therapeutic benefit for patients with GBM.

## 4 Conclusion

In this study, we demonstrated that thin GO nanosheets can be effectively non‐covalently complexed with the chemotherapeutic drug bortezomib which provided a pronounced tumor necrotic effect in vivo in two distinct orthotopic glioblastoma tumor models. These findings suggest that GO could be more widely applied as a delivery platform for chemotherapeutic drugs to achieve a higher local drug concentration when administrated locally. Overall, this work highlights a promising therapeutic strategy that can be used in combination with other therapies.

## 5 Experimental Section

### Reagents

Bortezomib (BTZ) was purchased from (Stratech, UK) with a purity of over 99.77%. Cell culture reagents and chemicals for the production of GO were purchased from Sigma‐Aldrich (Merck, UK) unless otherwise stated. Additional reagents used were obtained from commercial suppliers.

### Synthesis of Graphene Oxide (GO)

Biological‐grade GO was synthesized using a modified Hummers' method previously described. Briefly, graphite powder was mixed with sodium nitrate, followed by dropwise addition of sulphuric acid. Subsequently, potassium permanganate was added to the mixture. Then, water was added to the solution, keeping the temperature at 98 °C. After that, hydrogen peroxide was added to the mixture to stop the reaction. The purification of the GO was performed under endotoxin‐free conditions by several centrifugation and washing steps. GO sheets with lateral dimensions ranging from 25 nm to 1.9 µm were prepared by sonicating for 5 min and consequently purification to remove the largest debris. (Table S1, Supporting Information).

### Preparation of GO:BTZ Complexes

The complexation of GO and BTZ was performed through non‐covalent interactions between both entities. Initially, bortezomib was dissolved in non‐pyrogenic water containing 0.001% (w/w) acetic acid (Sigma‐Aldrich). Then, 1 mg ml^−1^ GO suspension was mixed with the BTZ solution at a 10:6 mass ratio (w/w) followed by incubation for 1 h under moderate agitation (1 G). Afterward, the solution was centrifuged for 50 min (21 000 g) at room temperature and washed three times with water to remove any unbound BTZ, and the obtained product was re‐dispersed in non‐pyrogenic water to obtain a homogenous and stable suspension. For biological experiments, following a single washing step as described above, the GO:BTZ complex was resuspended in either water for injection (Fannin, Greece) with 5% dextrose (Sigma‐Aldrich) for intratumoral injections, or water for cell culture (ThermoFisher, UK) and re‐suspended in cell culture medium for the in vitro experiments.

### Physicochemical Characterization of GO and GO:BTZ Complex

*Absorbance spectra* were acquired with a UV–vis–NIR Jasco V‐780 spectrophotometer, at the ICMAB Spectroscopy Facility, at room temperature using samples prepared in water dispersion. The accuracy of the absorbance of UV–vis spectrophotometer was tested using the certified UV–vis Standard 1 (potassium dichromate, 60.06 mg L^−1^ in sulphuric acid, 0.01N, Batch HC909064), complying with the European Pharmacopeia (Ph. Eur.) specifications. X‐Ray photoemission spectroscopy measurements (XPS) were obtained using a Phoibos 150 (SPECS, GmbH) electron spectrometer equipped with a hemispherical analyzer, at the ICN2 Photoemission Spectroscopy (XPS&UPS) Facility, operating under ultrahigh‐vacuum conditions, and with an Al K*α* (h*ν* = 1486.74 eV) X‐ray source. Samples were prepared using the drop‐casting method. The morphology of the materials deposited on freshly cleaved mica (Ted Pella) was determined by atomic force microscopy (AFM, Asylum MFP‐3D, Oxford instruments) in tapping mode and equipped with silicon probes (Ted Pella) with a resonance frequency of 300 kHz and a nominal force of 40 N m^−1^.

### Cell Lines and Cell Culture Conditions

Human glioma cell line U87‐MG and U87‐luc was obtained from the American type culture collection (ATCC) and were tested free from mycoplasma contamination. Cells were cultured in normal minimum essential medium eagle (MEM), with L‐glutamine, Earle's salts, (2.2 g L^−1^) sodium bicarbonate (added from the manufacturer), and 10% fetal bovine serum (FBS; Gibco, Thermo Fisher Scientific, UK) and 1% antibiotics (penicillin and streptomycin, PenStrep) at 37 °C, 5% CO_2_. The murine GL261‐luc cell line was kindly provided by Prof. Brian Bigger (The University of Manchester, UK) and was cultured in RPMI‐1640 medium supplemented with 10% FBS and 1% penicillin/streptomycin.

### LDH Cytotoxicity Assay

The release of lactase dehydrogenase (LDH) was measured by using a CytoTox96 nonradioactive cytotoxicity assay kit following the manufacturer's guidelines (Promega, G1780). Cells were seeded in 12‐well or 24‐well plates (Costar, UK) and allowed to reach 75% confluence before the medium was aspirated and replaced with treatments followed by incubation for 24 h at 37 °C with 5% CO_2_. Following incubation, cell supernatant was transferred to a 96‐well plate and centrifuged at 4000 rpm for 20 min. 50 µl of each conditioned media‐supernatant was transferred to a separate well in a 96‐well‐plate and an equal volume of substrate solution was added. Samples were incubated at room temperature for 20 min before measurement of the absorbance at *λ* = 492 nm using a Fluostar Omega microplate reader (BMG Labtech). Cytotoxicity was measured by the release of LDH and was expressed as the percentage viability relative to control based on *n* = 3 biological replicates per condition. Control conditions: 15% DMSO –positive control, untreated cells‐ negative control. Data are reported as a percentage release of LDH compared to the untreated control.

### Cell Counting Cytotoxicity Assay

As above, cells were seeded 1 day before treatment and allowed to reach 75% confluence. Following incubation with the different treatments, for 24 h, at 37 °C with 5% CO2, cells were detached using 0.05% trypsin‐EDTA for 5 min, followed by the addition of complete media containing 10% of FBS. The cell solution was then combined at a 1:1 ratio with 0.4% Trypan blue, and single live cells were counted using a haemocytometer‐ twice per sample with three biological replicates per condition. As controls, 15% DMSO and untreated cell samples were used. Data are reported as a percentage of live cells compared with untreated cells.

### Imaging

Cells were imaged using a PrimoVert microscope (ZEISS) using a Primo Plan‐ACHTOMAT 10X/0.25 Ph1 lens and images were captured via an AxioCam ERc5s camera with ZEN light software. Imaging conditions were kept consistent throughout.

### Animals

All animal experiments were performed at the University of Manchester (UK), in accordance with the Animals (Scientific Procedures) Act 1986 (UK), approved by the University of Manchester Ethical Review Committee and under a UK Home Office Project License P089E2EOA and following the ARRIVE 2.0 guidelines. Animals were housed in groups within ventilated cages with ad libitum access to food and water. Female athymic nude‐Fox1^nu^ (Envigo, UK) and C57/BL6 (Envigo, UK) mice were allowed to acclimatize to the facility for at least one week prior to any procedure.

### Intracranial Inoculation of Glioma Cells

Female athymic nude mice for the U87 model (6–8 weeks old) and female C57/BL6 mice (8–9 weeks old) were anesthetized using isoflurane (2.5% induction and 1–2% maintenance in medical oxygen, at a rate of 1.5 L min^−1^) and placed on a stereotactic frame. Prior to incision, animals received 0.1 mg kg^−1^ of buprenorphine (Buprenex, Reckitt Benckiser, UK). A midline incision was performed to expose the cranium and a 0.7 mm borehole was drilled (Fine Science Tools, Canada) above the right striatum at 0.0 mm anterior and 2.3 mm lateral from bregma. A 10 µl Hamilton syringe (SYR10, Hamilton, USA) fitted with a 26‐gauge blunt needle (Hamilton, USA) was lowered to 3 mm below the cortical surface and slowly withdrawn 0.6 mm such that the injection took place at 2.4 mm depth. 1 × 10^5^ U87‐luc cells or 5 × 10^4^ GL261‐luc cells in 1 µl of PBS were injected slowly over 5 min at a rate of 0.2 µl min^−1^. Post‐injection the needle was kept in place for 3 min to minimize reflux and slowly withdrawn over 1 min to minimize any injury. The skin incision was closed with 5‐0 coated vicryl sutures (Ethicon, UK) and animals were allowed to recover in a heated environment.

### Intratumoral Injection

Mice underwent intratumoral injection with 1 µl vehicle (5% dextrose), GO (0.9–1 µg), BTZ (0.175 µg), or GO:BTZ (containing 0.175 µg BTZ) in 5% dextrose at the time specified in the figure legend for each experiment. Mice were anesthetized and prepared for stereotaxic surgery as described above. The original incision was reopened and a 33‐gauge needle connected to a 10 µl Hamilton Neuros syringe was passed through the original borehole to a depth of 2.4 mm. 1 µl of material or vehicle control was injected over 5 min (0.2 µl min^−1^). Post‐injection the needle was kept in place for 3 min to minimize reflux and slowly withdrawn over 1 min. The skin incision was closed with 5‐0 coated vicryl sutures and animals were allowed to recover in a heated environment.

### In Vivo Bioluminescence Imaging (BLI)

Tumor‐bearing mice were anesthetized with 1.5% isoflurane followed by intraperitoneal injection of 150 mg kg^−1^ mouse D‐luciferin (15 mg ml^−1^; Promega, UK) in PBS. After 8 min, bioluminescence signals were detected using sequential imaging (10 measurements at 2 min intervals) with an in vivo imaging system (IVIS Lumina II, PerkinElmer, UK). Images were analyzed with Living Image software (version 4.7) (PerkinElmer, UK).

### MRI Acquisition and Analysis

MRI was conducted using a 7 Tesla magnet connected to a Bruker Advance III console (Bruker Biospin Ltd, UK). Mice were placed in a magnet capsule with a cylindrical surface coil and imaged under isoflurane anesthesia (3% induction and 1–2% maintenance in medical oxygen at a rate of 1.5 L min^−1^). The respiratory rate was monitored throughout imaging and anesthetic level was controlled based on the respiratory parameters (50–70 bpm). After localizing imaging on three orthogonal axes, the whole brain was imaged using T2 weighted MRI: Rapid Acquisition with Relaxation Enhancement (RARE) pulse sequence, repetition time = 2200 ms, echo time 33 ms, RARE factor 8, the field of view (FOV) 30 × 30 mm matrix size 512 × 512, 17 contiguous slices, thickness 0.8 mm, averages = 5. Tumor volumes were determined using MRIcron software (National Institutes of Health, NIH, Bethesda, USA). The tumor area was delineated for each coronal slice and the total tumor volume was generated from the acquired measurements.

### Tissue Processing and Staining

At the end of each experiment, tumor‐bearing mice were anesthetized with 2.5% isoflurane and culled by cardiac perfusion with 2 mM EDTA in PBS, followed by 4% PFA in PBS. Brains were removed and fixed overnight at 4 °C and later placed in 30% sucrose in PBS for at least 24 h. The brains were snap‐frozen in cold isopentane (−40 °C) and coronal sections (20 µm thickness) were taken using a cryostat (Leica CM1950, Leica Biosystems, Germany).

Sections were stained with haematoxylin and eosin (H&E) staining to observe the histological characteristics of the tumor sections and determine the tumor volume. Cryosections were left for 15 min to air dry before fixation with pure ethanol for 2 min. Slides were washed once with PBS for 5 min, and were placed in haematoxylin for 1 min. Slides were washed twice with water for 3 min each and were placed in 70% EtOH for 3 min. Following dehydration, slides were placed in eosin solution (1% eosin in 95% alcohol) for approximately 40 s. This was followed by three washes in 100% alcohol for 3 min each, and slides were placed in two changes of Xylene for 2 min each. Finally, DPX mount was used to mount coverslips and slides were then left to dry overnight at room temperature. Slides were scanned using a 3D Histech Panoramic 250 slide scanner.

### Histological Evaluation of Tumor Growth

H&E stained sections were imaged using a Panoramic 250 slide scanner (3D Histech, Hungary) and analyzed using 3DHISTECH Case Viewer software version 2.6. Initially, tumor diameter was measured in each section so as to identify the maximal tumor area. Subsequently, the height and width of the tumor area were measured and the volume was calculated using the following formula:

(1) $$V=(W^{2}\times H)/2$$

*The above formula was chosen after the comparison with V = (W × H × L)/2 where length indicates the tumor thickness based on the slices cut and slice thickness.

### Raman Mapping

U87 brain cryosections were used for Raman Spectroscopy imaging in which GO was identified by a Raman spectrum of two distinct D and G bands at 1352 and 1594 cm^−1^, respectively, and a less projecting 2D band at 2707 cm^−1^. That spectrum was used as a fingerprint to validate the presence of GO in the tumor area. Raman mapping was performed using a HORIBA XploRA Raman microscope working with a 638 nm laser operating at 25% of power, passed through a 300 µm hole and a 100 µm slit. A 50x objective was used to collect the Raman spectrum at each single pixel of the region of interest.^[^ ^27^ ^]^

### Immunofluorescence (IF) Staining

For immunofluorescence analysis, 20 µm cryo‐sections samples were air‐dried and fixed for 10 min in ice‐cold acetone. After washing the samples with PBS, sections were incubated for 1 h with 1% bovine serum albumin and 5% donkey serum in PBS‐Triton X 0.2% to remove any non‐specific binding. Rabbit anti‐mouse IBA1 antibody (dilution 1:1000, Fujifilm, 019–19741 Wako) was incubated overnight at 4 °C, for staining activated macrophage and microglia cells. For secondary antibody staining, Alexa 488‐conjugated donkey anti‐rabbit IgG (dilution 1:1000, Abcam, ab150073) was used. Sections were washed and Prolong Gold medium with DAPI was added and covered with coverslips. Images were taken with a Histec Pannoramic250 slide scanner.

### Statistical Analysis

Statistical analysis was performed using GraphPad Prism 8.1.2 (332). For the comparison of two groups, an unpaired Student's *t*‐test was used and for comparison of three or more groups, an ordinary one‐way ANOVA (Tukey's multiple comparison test) or two‐way ANOVA (Tukey's and Sidak's multiple comparisons test) were utilized. Data was regarded as statistically significant if *p* < 0.05. *p‐*values and statistical tests are specified in the figure legend for each data. Data are presented as mean ± SD or S.E.M as defined in the figure legend of each figure.

## Conflict of Interest

The authors declare no conflict of interest.

## Author Contributions

P.S.S. and M.S. contributed equally to this work. K.K. and P.S. initiated and designed the study. K.K. and T.K. coordinated the study. P.S. conducted most of the in vivo experiments and in vivo imaging. M.S. assisted in all the in vivo experiments and performed the in vitro experiments. T.K. performed in vivo experiments, imaging, and data analysis. A.M.G., L.M.A., J.C.N., and N.L. developed the complexation protocol and performed the characterization of the complex. A.D. performed tissue processing and assisted in image analysis. K.B. performed bioinformatics analysis for the quantification of the necrotic tissue and nanomaterial distribution. M.S., T.K., P.S., L.M.A., N.S., and K.K. contributed to the writing of the manuscript.

## Supporting information

Supporting Information

## Data Availability

The data that support the findings of this study are available in the supplementary material of this article.
