# Supplementary material for: Graphene Oxide Nanoscale Platform Enhances the Anti‐Cancer Properties of Bortezomib in Glioblastoma Models
Source: Adv Healthc Mater. 2022 Nov 11;12(3):2201968. doi: 10.1002/adhm.202201968 (PMC11468189; doi:10.1002/adhm.202201968)
Supplement: Supplementary file 1 — Supporting Information [file ADHM-12-2201968-s001.pdf]

ADVANCED HEALTHCARE MATERIALS

Supporting Information

for *Adv. Healthcare Mater.*, DOI 10.1002/adhm.202201968

Graphene Oxide Nanoscale Platform Enhances the Anti-Cancer Properties of Bortezomib in Glioblastoma Models

Paul S. Sharp, Maria Stylianou, Luis M. Arellano, Juliana C. Neves, Alfredo M. Gravagnuolo, Abbie Dodd, Katharine Barr, Neus Lozano, Thomas Kisby and Kostas Kostarelos**

SUPPORTING INFORMATION

Graphene oxide nanoscale platform enhances the anti-cancer properties of bortezomib in glioblastoma models

Paul S. Sharp[1,^,#], Maria Stylianou[1,#], Luis M. Arellano[2], Juliana C. Neves[2], Alfredo M. Gravagnuolo[1], Abbie Dodd[1], Katharine Barr[1], Neus Lozano[2], Thomas Kisby[1*], Kostas Kostarelos[1,2*]

Supplementary Experimental

Supplementary physicochemical characterization of GO and GO:BTZ complex. *Scanning electron microscopic (SEM)* images were performed using a FEI Magellan 400L filed emission microscope (Oxford instruments) equipped with an Everhart-Thornley as secondary electrons detector and using an acceleration voltage of 20 kV and beam current of 0.1 nA, at the ICN2 Electron Microscopy Unit. Samples were deposited on an Ultrathin C on Lacey C grid (Ted Pella). *Raman Spectroscopy*. Raman spectra were acquired with a confocal Raman microscope (Witec) equipped with a 632 nm laser excitation and using a gradient of 600 g/nm. *X-Ray diffraction (XRD)* measurements were carried out using a diffractometer (Malvern PANalytical X'pert Pro MPD) equipped with a ceramic X-ray tube with Cu Kα anode (λ=1.540 Å) as x-ray source and x'Celerator solid-state detector in the 2θ scan range from 5° to 40°, at the ICN2 X-Ray Diffraction Facility. *Fourier transform infrared (FTIR)* spectra were recorded on a Tensor 27 FT-IR spectrometer (Bruker) by casting the sample on potassium bromide within a 4 cm-1 resolution and scan range of 3800-600 cm-1, at the ICN2 Molecular Spectroscopy and Optical Microscopy Facility. *Zeta potential and the hydrodynamic diameter* of the different samples were also measured by a Zeta-sizer Nano ZS (Malvern instruments) equipped with disposable capillary cells, at the ICN2 Molecular Spectroscopy and Optical Microscopy Facility.

Image analysis and classification. Histological images were analysed using Orbit classification software as described by the developer's**.**[66] The model was trained by manual classification of the three regions of interest (normal brain, glioblastoma tissue, necrotic tissue) across 3 individual samples. For quantitative analysis, the trained model was used to classify these regions across 2 sections from 4 mice per treatment group and number of pixels for each classification recorded. Data was presented as necrotic area as a % of total glioblastoma area (necrotic + glioblastoma).

Supporting Figures

Supporting Figure 1

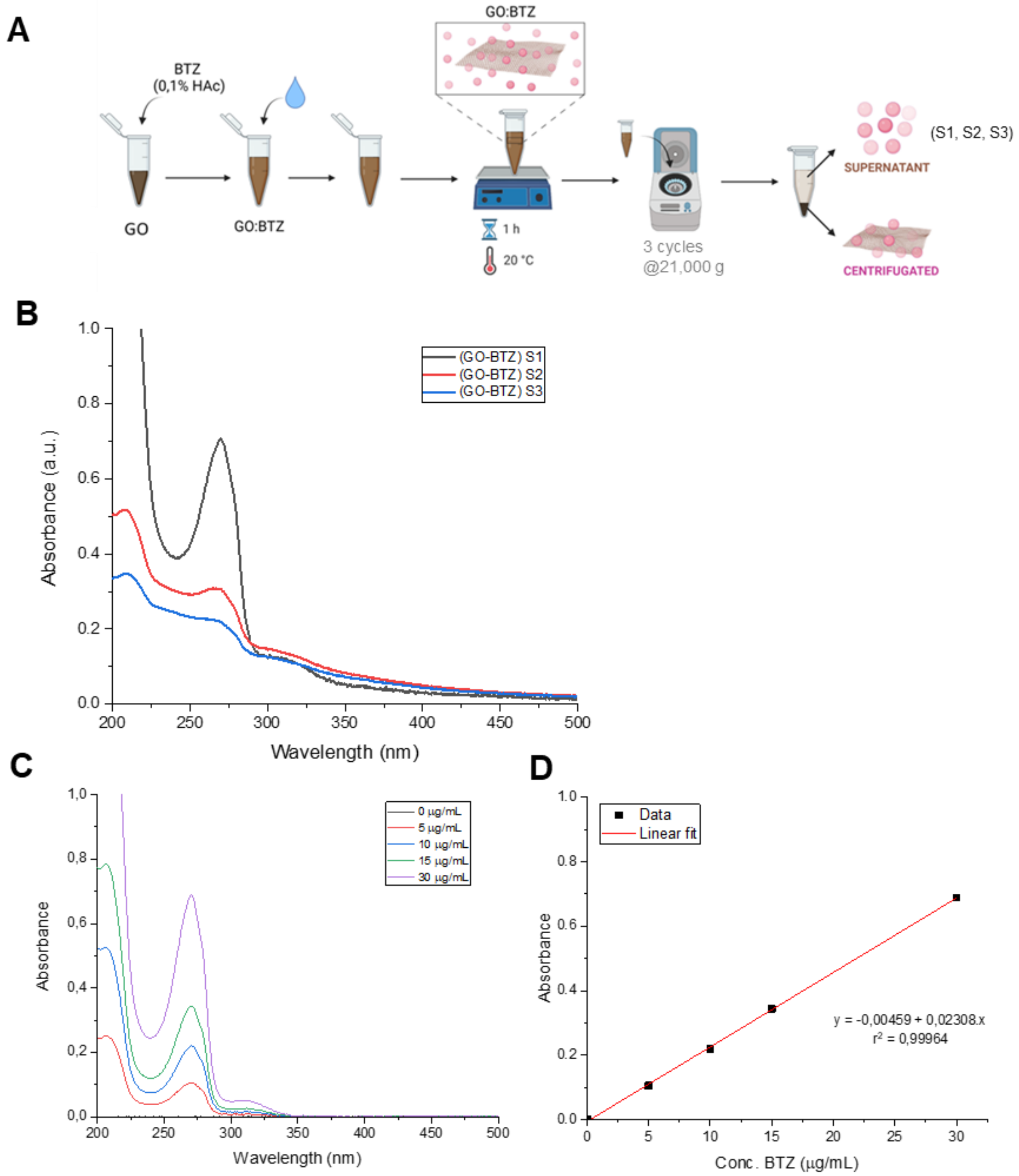

Figure S1. Non-covalent GO:BTZ complex formation and determination of BTZ loaded in complex. (**A**) Graphical representation of the step-wise protocol used for the GO:BTZ complex formation. (**B**) UV-vis spectrum for supernatants (S1; S2; S3) obtained following 3 sequential washing steps by centrifugation and resuspension in water. The sum of this (unbound) was used to indirectly quantify the amount of BTZ bound to GO in the complex. (**C**) UV-vis spectrum of free BTZ in water at different concentrations. (**D**) Standard curve for BTZ in water based on 270nm peak used for quantification.

Supporting Figure 2

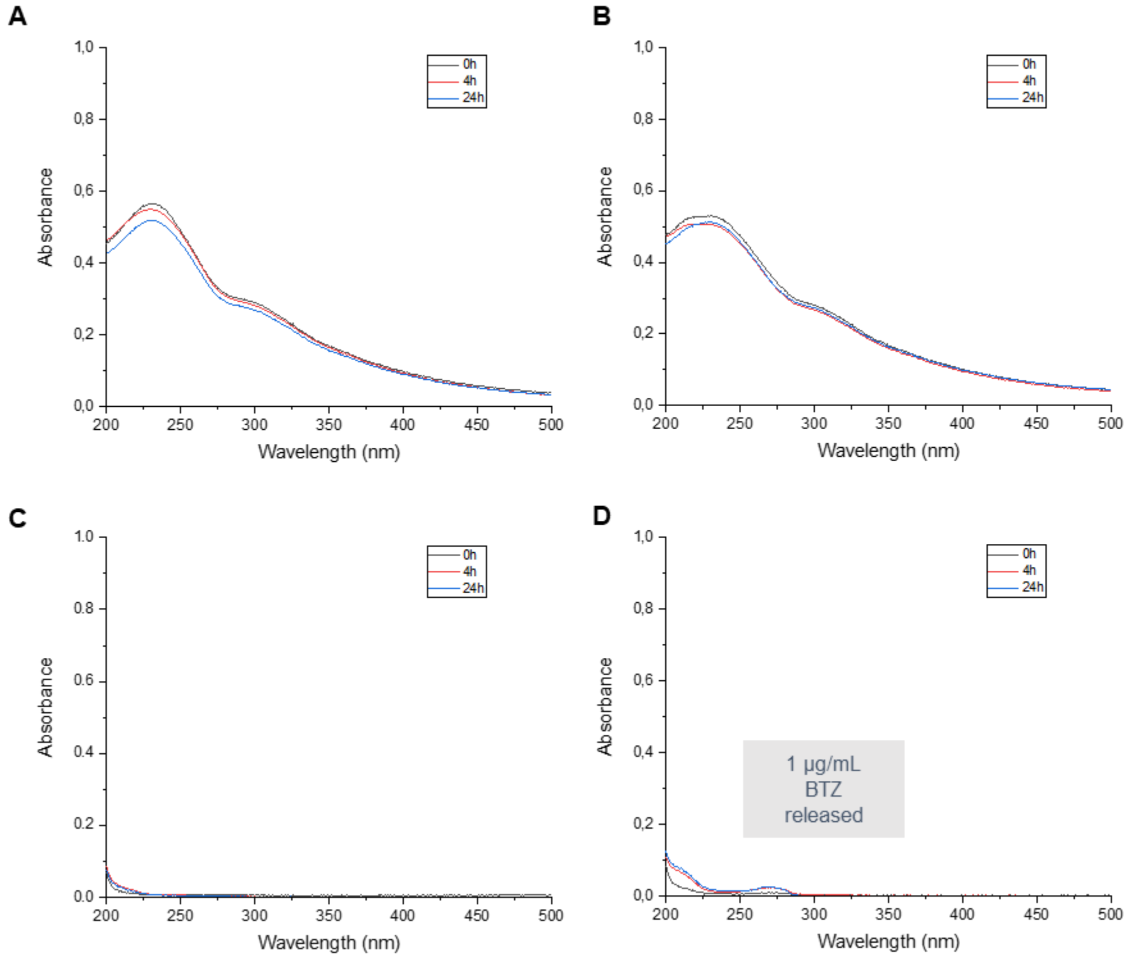

Figure S2. BTZ release profile in water from GO:BTZ complex measured by UV-vis after 0h, 4h or 24h in water. (**A**) GO control; (**B**) GO:BTZ complex; and their corresponding supernatant (relating to released BTZ) fractions (**C**) and (**D**), respectively.

Supporting Figure 3

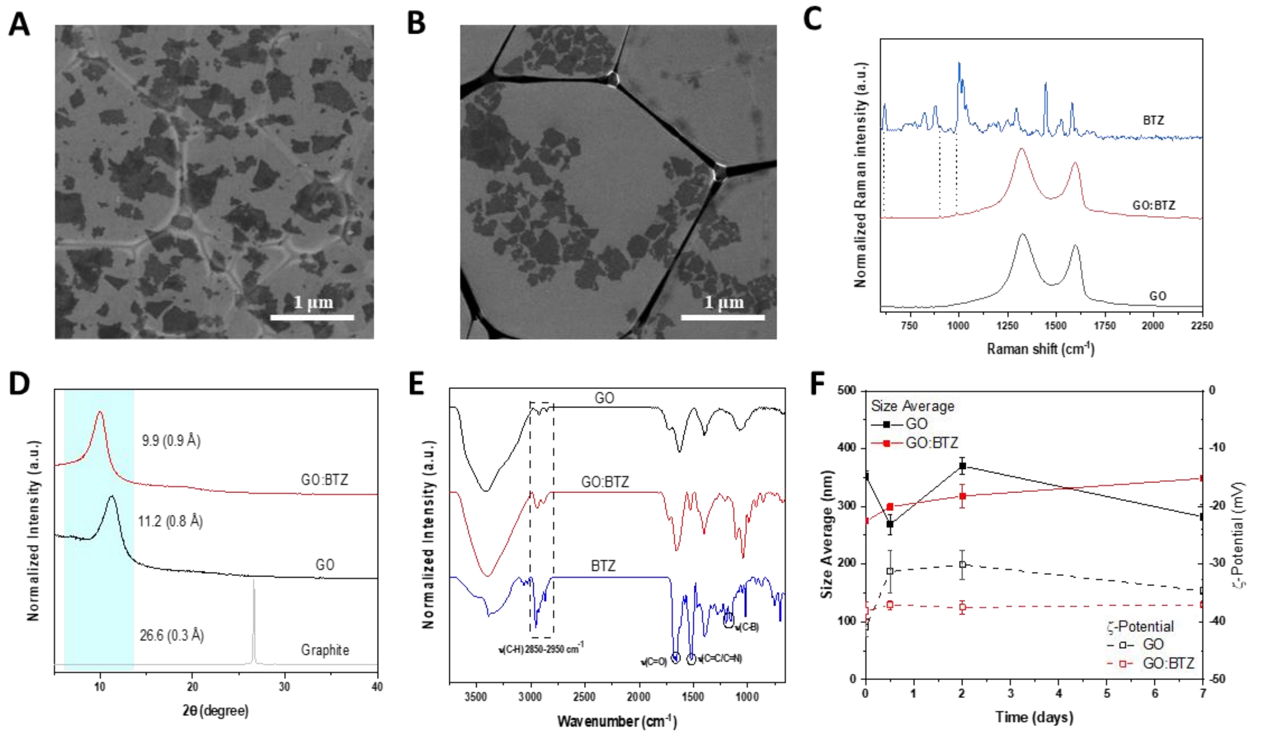

Figure S3. Physicochemical characterization of GO_c and GO:BTZ complex. SEM micrographs of (**A**) GO_c and (**B**) GO:BTZ complex. (**C**) Normalized Raman spectra. **(D)** XRD patterns of starting graphite, GO_c and GO:BTZ complex. GO peaks at 3400, 1734, 1633 and 1074 cm-1. (**E**) Fourier-transform infrared spectra of GO_c, BTZ and GO:BTZ complex. (**F**) Size average (solid symbols, straight lines) and ζ-potential values (open symbols, dotted lines) over 7 days. Average hydrodynamic diameter ~300nm and a low polydispersity index (0.3).

Supporting Figure 4

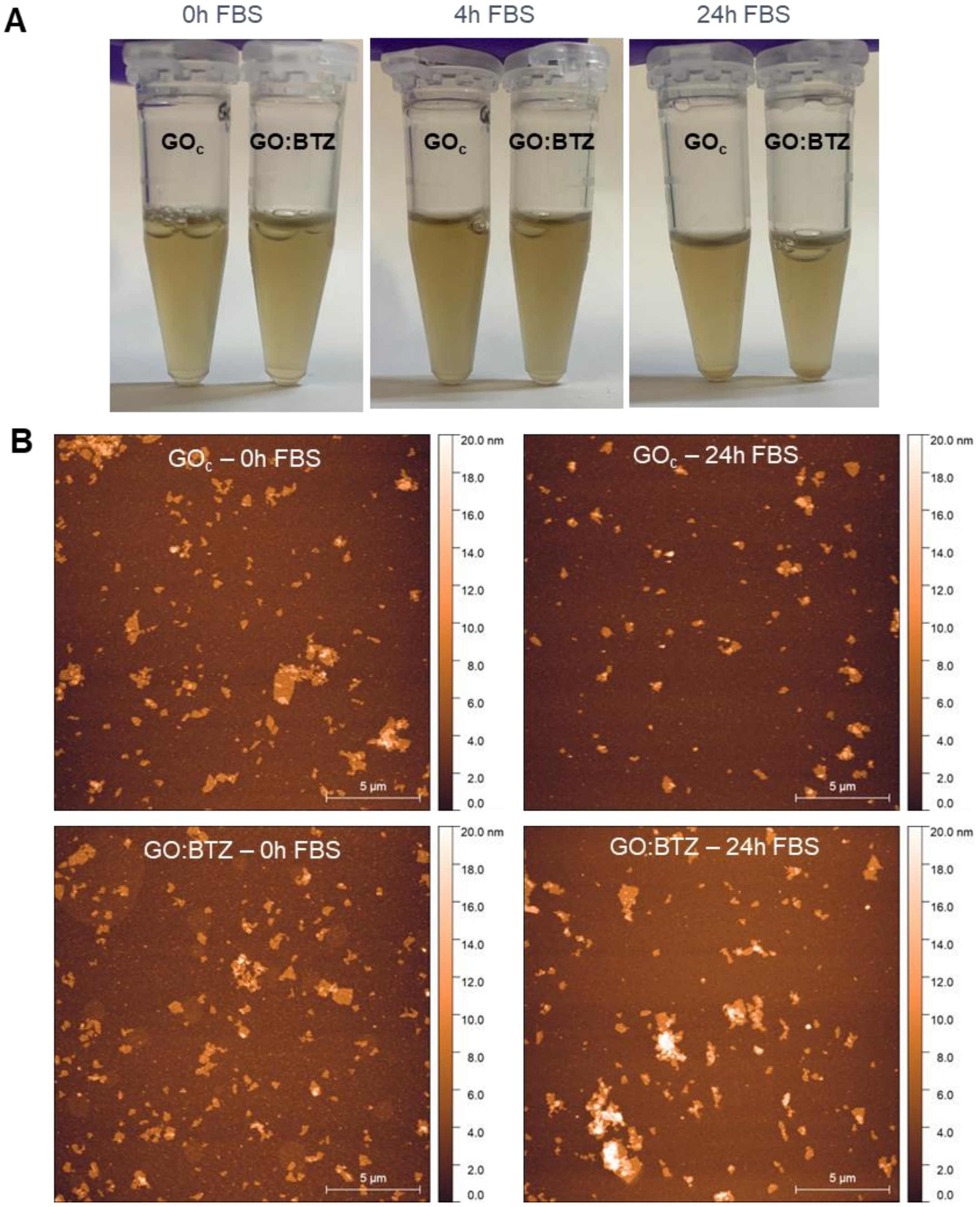

Figure S4. GO and GO:BTZ nanosheet suspension characteristics in serum (10% FBS) for 24hrs. (**A**) Photographs of GO_c and GO:BTZ samples after 0 hours, 4 hours and 24 hours following dilution in 10% FBS; (**B**) Height Atomic force microscopy (AFM) images for GO_c and GO:BTZ complex after 0h and 24h in 10% FBS (scale bar is 5 µm). All samples were diluted in 10% FBS to reach a final GO concentration of 0.1 mg/ml.

Supporting Figure 5

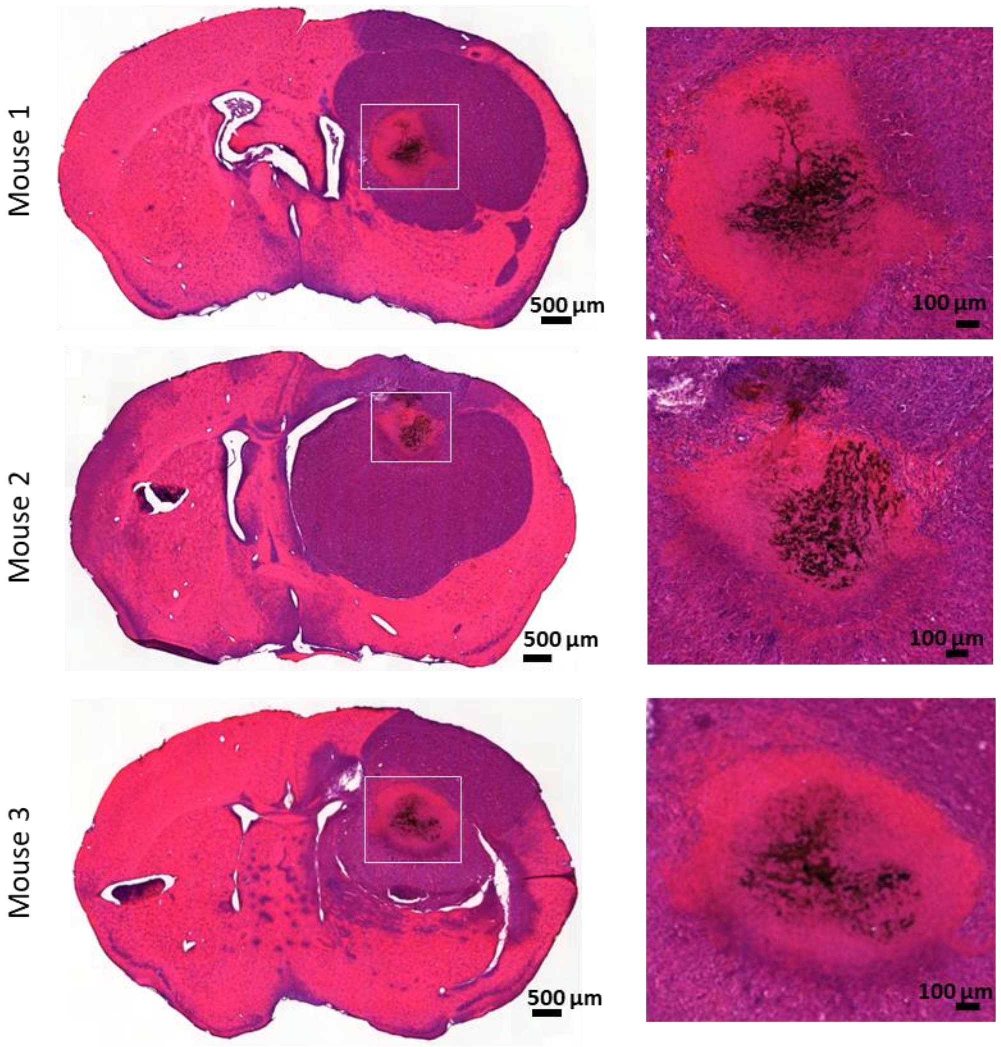

Figure S5. GO-BTZ forms a necrotic zone extending from the GO injection site. Representative H&E images showing the effect of GO:BTZ; n=3) at 12 days post- intratumoral injection (i.t.). Each image shows an individual mouse highlighting the localisation of the necrosis to the initial site of injection. Scale bars, 500 µm and 100 µm.

Supporting Figure 6

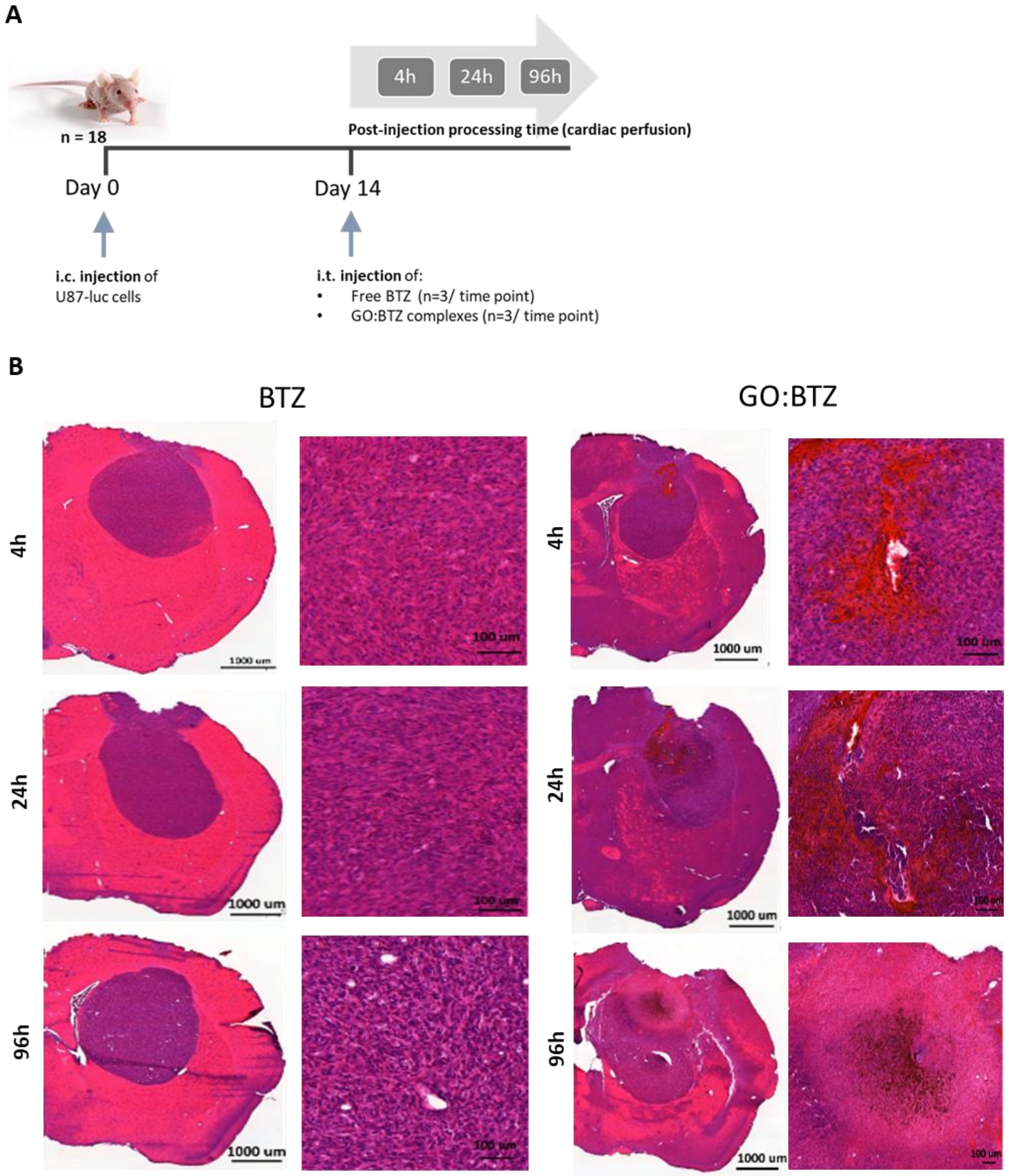

Figure S6. Kinetics of GO:BTZ induced necrosis with direct comparison to free BTZ. (**A**) Experimental schematic for the *in vivo* analysis. Athymic nude mice were implanted with $1x10^5$ (1µl) U87-luc cells into the right striatum. Histological analysis was performed at 4 h, 24 h and 96 h following intratumoral (i.t.) delivery of free BTZ or GO:BTZ (n=3 / timepoint). (**B**) Representative H&E stained sections at 4h, 24h and 96h post treatment. Scale bars, 1000 µm and 100 µm.

Supporting Figure 7

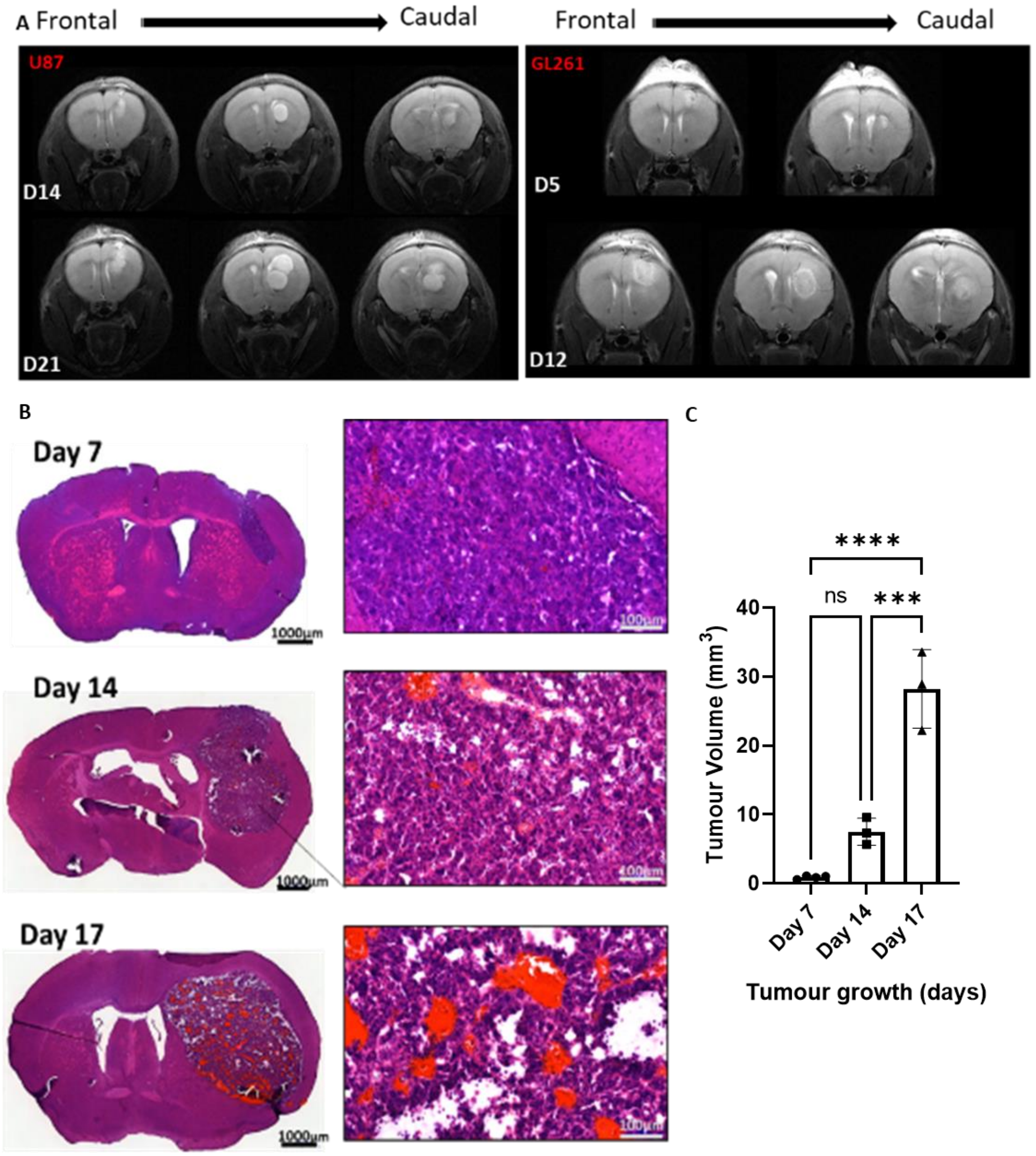

Figure S7. Characterisation of the U87 and GL261 tumor models. (**A**) Coronal T_2-weighted MRI images on day 14 and day 21 following i.c. injection of $1x10^5$ (1µl) U87-luc cells in athymic mice. Coronal T_2-weighted MRI images on day 5 and day 12 following i.c. injection of $5x10^4$ (1µl) GL261-luc cells in C57BL/6 mice. (**B**) Histological analysis of GL261 tumors was performed at 7, 14 and 17 days post inoculation showing more heterogenous features. Scale bars, 1000 µm and 100 µm. (**C**) Volumetric quantification of tumor from histology at day 7, day 14 and day 17 of GL261-luc tumor growth. Data presented as mean ± S.D. Ordinary one-way ANOVA, Tukey's multiple comparisons test (***p≤0.001, ****p ≤ 0.0001).

Supporting Figure 8

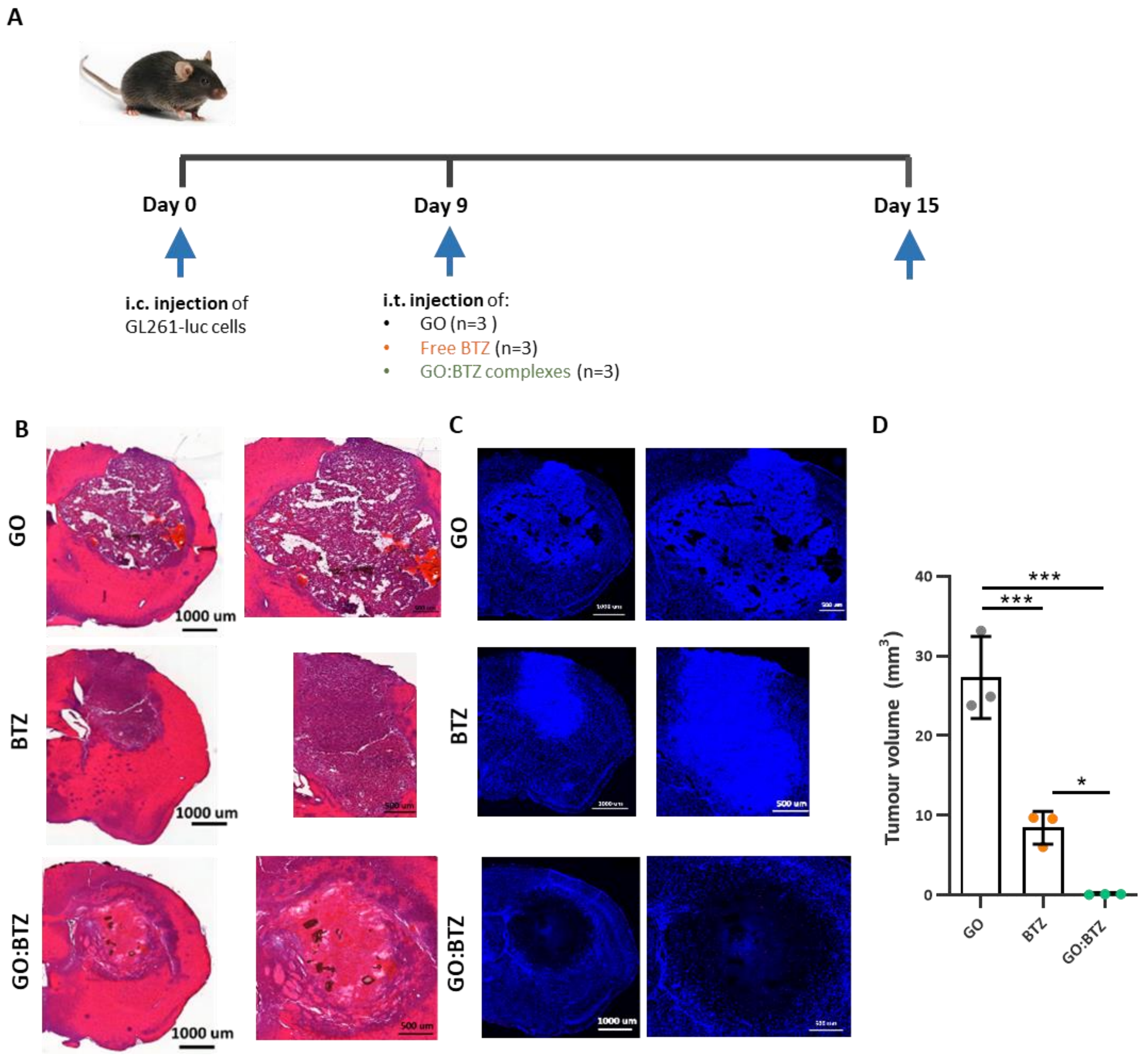

Figure S8. GO-BTZ forming a significant necrotic core within the injected tumor area. (**A**) Experimental schematic for the *in vivo* analysis. C57BL/6 mice were implanted with $5x10^4$ (1µl) GL261 cells into the right striatum. (**B**) Representative H&E images performed 6 days following intratumoral (i.t.) delivery of GO_c, free BTZ or GO-BTZ (Scale bars, 1000 µm and 500 µm). (**C**) Representative images DAPI (blue); on fixed tissue 6 days following intratumoral (i.t.) delivery of GO_c, free BTZ or GO-BTZ. Scale bars, 1000 µm and 500 µm. (**D**) Quantification of tumor volume based on the H&E analysis. Data presented as mean ± S.D. Ordinary one-way ANOVA-Tukey's multiple comparisons test (*p≤0.05, ***p≤0.001).

Supporting Figure 9

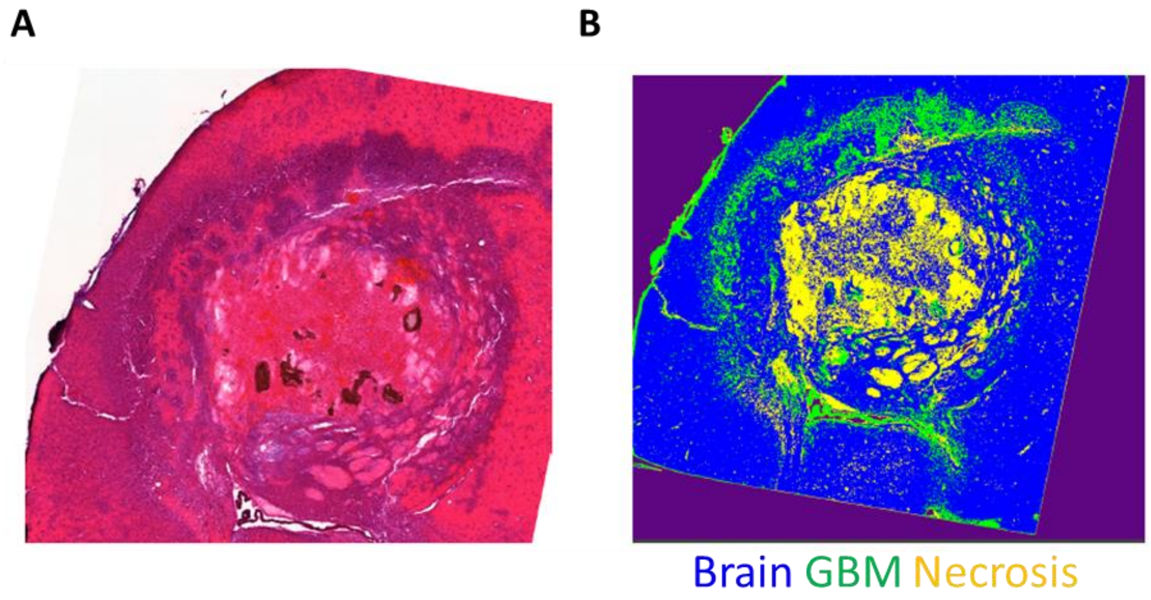

Figure S9. Representative Orbit image classification. (**A**) The model was trained by manual classification of H&E images to identify normal brain, GBM tissue and areas of necrosis (devoid of any cellular structures/nuclei) over at least 3 independent samples. (**B**) Trained model output when analysing a previously unclassified image.

Supporting Figure 10

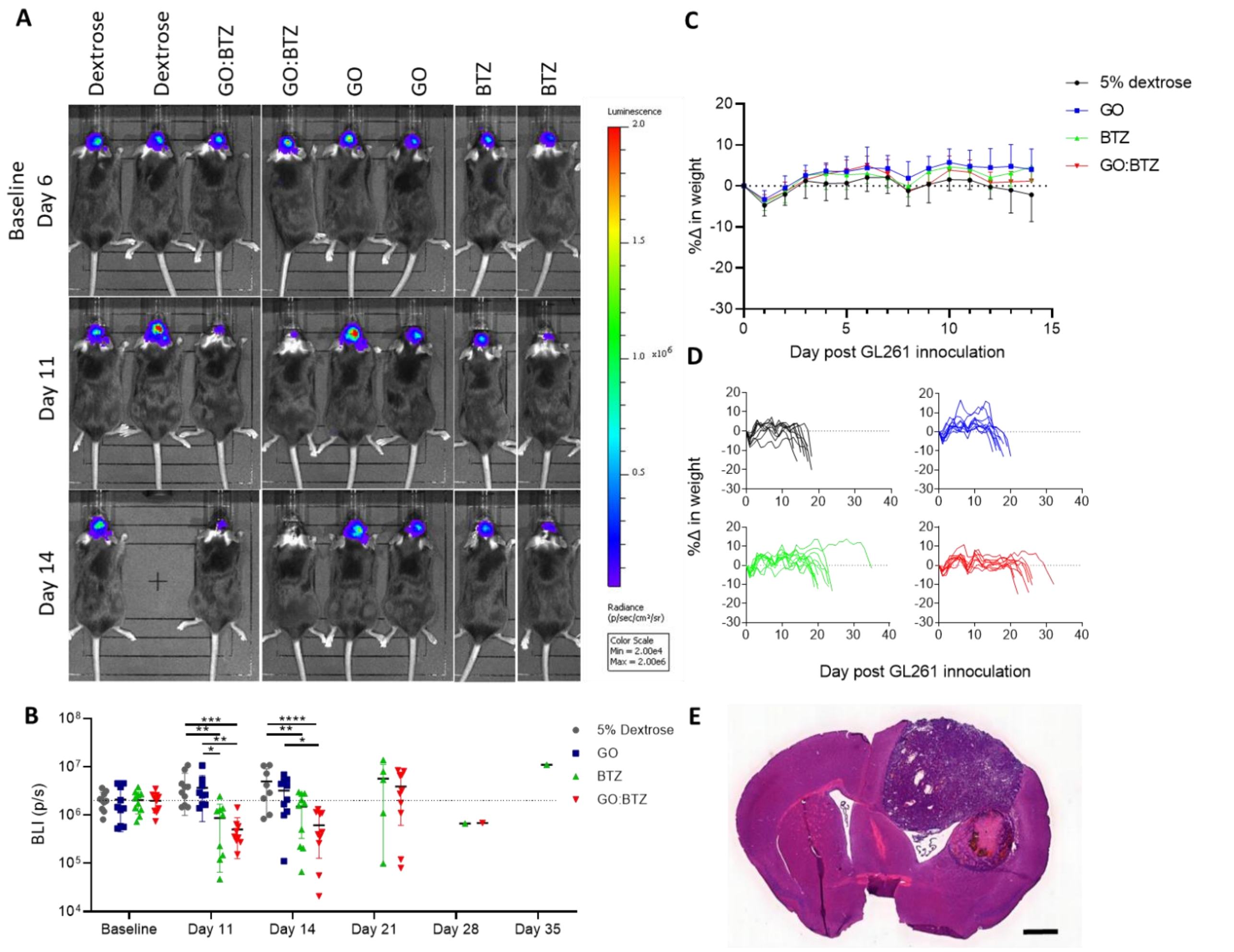

Figure S10. Longitudinal assessment of treatment effect and biosafety. (**A**) Representative IVIS imaging of mice before (day 6) and after (day 11 and 14) treatment. (**B**) Quantification of bioluminescence (BLI) signal relative to the pre-treatment baseline across all treatment groups. Data presented as mean ± S.D, Two way ANOVA with Tukey post-hoc analysis. *, **, ***, **** represents p<0.05, p<0.01, p<0.001 and p<0.0001 respectively. (**C**) Animal weight presented as percentage change (Δ) from starting weight. Mean ± S.D shown for each group during acute treatment phase and (**D**) each animal plotted individually throughout the entire study duration. (**E**) Representative histology of mice treated with GO:BTZ complex at sacrifice (day 25) showing superficial (cortical) tumor growth above treatment site (striatum), scale bar = 1000 µm.

Supporting Tables

Supporting Table 1

Table S1: Physicochemical properties of GO sheets including lateral dimension, thickness, optical properties, surface charge and chemical composition.

	Technique	GO
Physicochemical properties		
Lateral dimension	SEM	50 nm - 1.9 µm<br>95% < 850 nm<br>Mean 332 nm<br>[n=624]
	AFM	25 nm - 1.5 µm<br>95% < 475 nm<br>Mean 87 nm<br>[n=1426]
Thickness	AFM	1 - 2 nm
Optical properties	Absorption spectroscopy	ε_{230} (mL µg^{-1} cm^{-1})= 0.053
	Fluorescence λ_{600} (λ_{exc525})	0.964 * C_{GO} (µg/mL)
Degree of defects (I_D/I_G)	Raman spectroscopy	1.14 ± 0.03
Peak (2θ)	XRD	12.4 °
Interlayer distance (nm)		0.7
Surface charge	Electrophoretic mobility	-52.1 ± 0.4 mV
Chemical composition	XPS	C: 72.2%, O: 25.0 %, S: 1.2%, B: 1.6%,
Purity (%C + %O)		97.2%
C:O ratio		2.9
